# A novel model incorporating quantitative contrast-enhanced ultrasound into PI-RADSv2-based nomogram detecting clinically significant prostate cancer

**DOI:** 10.1038/s41598-024-61866-x

**Published:** 2024-05-15

**Authors:** Kaifeng Huang, Li Luo, Ruixia Hong, Huai Zhao, Ying Li, Yaohuang Jiang, Yujie Feng, Qihuan Fu, Hang Zhou, Fang Li

**Affiliations:** 1 Department of Ultrasound, Chongqing University Cancer Hospital, Chongqing, China; 2 Chongqing Key Laboratory for Intelligent Oncology in Breast Cancer (iCQBC), Chongqing University Cancer Hospital, 181 Hangyulu, Shapingba, Chongqing, 400030 China; 3 Chongqing University Cancer Hospital, School of Medicine, Chongqing University, Chongqing, China

**Keywords:** Quantitative contrast-enhanced ultrasound, Prostate imaging reporting and data system, Nomogram, Clinically significant prostate cancer, Cohort study, Cancer imaging, Cancer screening, Urological cancer

## Abstract

The diagnostic accuracy of clinically significant prostate cancer (csPCa) of Prostate Imaging Reporting and Data System version 2 (PI-RADSv2) is limited by subjectivity in result interpretation and the false positive results from certain similar anatomic structures. We aimed to establish a new model combining quantitative contrast-enhanced ultrasound, PI-RADSv2, clinical parameters to optimize the PI-RADSv2-based model. The analysis was conducted based on a data set of 151 patients from 2019 to 2022, multiple regression analysis showed that prostate specific antigen density, age, PI-RADSv2, quantitative parameters (rush time, wash-out area under the curve) were independent predictors. Based on these predictors, we established a new predictive model, the AUCs of the model were 0.910 and 0.879 in training and validation cohort, which were higher than those of PI-RADSv2-based model (0.865 and 0.821 in training and validation cohort). Net Reclassification Index analysis indicated that the new predictive model improved the classification of patients. Decision curve analysis showed that in most risk probabilities, the new predictive model improved the clinical utility of PI-RADSv2-based model. Generally, this new predictive model showed that quantitative parameters from contrast enhanced ultrasound could help to improve the diagnostic performance of PI-RADSv2 based model in detecting csPCa.

## Introduction

Prostate cancer (PCa) is a common malignant tumor in males in western countries^1^. In the year of 2024, there were 299,010 estimated new cases of PCa and 35,250 estimated deaths in the United State, ranking 1st in estimated new cases and 2nd in estimated deaths among men respectively^2^. In China, according to the latest published authoritative statistics in the year of 2016^3^, the mortality rate of PCa is 33.6 in every 100,000 Chinese male and the incidence of PCa is 78.3 in every 100,000 Chinese male. In 2019, the age-standardized incidence rate of PCa is 17.3 in every 100,000 Chinese males, with a 95.2% rise compared to the year of 1990, which is not an optimistic sign while the growth rate of age-standardized incidence rate over the same period globally is 13.2%^4^. Though the incidence of PCa is rising rapidly, there is still a large proportion of patients with PCa diagnosed at autopsy would never have any clinical symptoms during their lifetime, and the 10-year survival rate of whom is much higher than patients with clinically significant symptoms^5^. These lesions in the patients are referred to as non-clinically significant prostate cancer (Non-csPCa), and misdiagnosis of clinically significant prostate cancer (csPCa) enlarges the underlying shortage of benefit as well as unnecessary payment and harm from medical treatment like surgery or radiation.

In the diagnostic landscape of prostate cancer, prostate specific antigen (PSA) and PSA-derived markers, including free PSA (fPSA), free-to-total PSA ratio (f/t), prostate specific antigen density (PSAD), PSA velocity, et al., had been proposed in different guidelines and consensuses^6,7^. Although many studies had shown that these indicators improved the accuracy of PCa detection, it’s still suboptimal and insufficient for early and accurate PCa diagnosis, especially in the differentiation between csPCa and Non-csPCa lesions^8^.

Another preferred diagnostic method is prostate imaging reporting and data system (PI-RADS)v2 criteria based on multi-parameter magnetic resonance imaging (mp-MRI)^9^, which has been the standard radiological stage criteria for prostate lesions with outstanding diagnositic performance. However, PI-RADSv2 is imperfect for large intra-observer variability and low positive predictive value^10^. Moreover, there is no definite probability of risk in PI-RADSv2 because it is a semi-quantitative scoring system. Therefore, a more accurate quantitative scoring system is required. One recently published study^11^ combining quantitative dynamic contrast-enhanced MRI and PI-RADSv2 had proved that quantitative apparent diffusion coefficient (ADC) values significantly improved the diagnostic accuracy of PI-RADSv2 in grade 3 and grade 4 lesions, indicating that incorporating quantitative imaging parameters into the application of PI-RADSv2 criteria may improve the diagnostic accuracy of csPCa. Another study^12^ combined enhancement patterns of contrast enhanced ultrasound and PI-RADSv2 to detect csPCa, the result showed good discriminative performance of the lesions in the patients with PSA ranged from 4 to 10 ng ml^−1^ and could improve pre-biopsy risk stratification in males with “gray zone” PSA level. Nevertheless, there is no research focusing on the role of quantitative ultrasonic parameters in the process of quantification of PI-RADSv2 criteria while transrectal ultrasound has been an important imaging modality for preliminary screening detection of csPCa and guiding method of lesion biopsy.

Quantitative contrast enhanced ultrasound (qCEUS) via VueBox®(Bracco, Italy) is a quantitative imaging modality to detect csPCa lesions. The principal of CEUS is based on the visualization of tumor neovascularization, which is an indispensable stage for the formation, survival, and metastasis of csPCa^13^. The functional imaging could be objectively interpreted into quantitative data by the dedicated post-processing software named VueBox® for analysis^14^. qCEUS is totally different from mp-MRI, qCEUS via VueBox® has been applied in many organs including liver^15^, prostate^16^, colon^17^ et al. Meanwhile, CEUS is a more convenient and economical imaging modality compared to dynamic contrast-enhanced MRI. There have been newly published article claiming that the scoring system based on the imaging characteristics of CEUS is similar to PI-RADS score in detecting PCa and csPCa^18^. Nevertheless, it is not clear whether the quantitative parameters of CEUS are helpful when incorporated into PI-RADSv2-based nomogram for the detection of csPCa.

This study aims at exploring the diagnostic accuracy of csPCa when qCEUS is combined with PI-RADSv2 and clinical indicators through clinical predictive model, and visualize the model by constructing a nomogram.

## Patients and methods

### Patients selection

The study was performed at the Department of Ultrasound Medicine of Chongqing University Cancer Hospital and patients diagnosed as prostate cancer from 2019 January to 2022 March were included. The study conformed to the Declaration of Helsinki, and was authorized by the institutional ethics committee of Chongqing University Cancer Hospital (No. 2019 (177)), and waived the requirement for the written informed consent of the patients because the selected clinical and imaging data in this retrospective study would not affect the prognosis and privacy of the patients.

The inclusion criteria were as followed: (1) the patients were diagnosed as prostate cancer pathologically; (2) underwent mp-MRI and TDCE-US examination; (3) detailed clinical and biochemical data obtained, including age,serum total prostate-specific antigen (TPSA), free prostate-specific antigen (FPSA), free-to-total prostate-specific antigen ratio (f/t), prostate specific antigen density (PSAD) and Gleason score in pathological report.

The exclusion criteria were as followed: (1) clinical data or laboratory tests were not complete; (2) pathological results were not complete (3) lack of Transrectal digital contrast enhanced ultrasound (TDCE-US), mp-MRI examination.

The pathological diagnostic criteria for clinically significant prostate cancer were (1) Gleason Score ≥ 3 + 4; (2) and/or tissue volume ≥ 0.5 cm^3^; (3) and/or extraprostatic invasion, these criteria are in consistent to that of PI-RADSv2 guideline^9^

### Transrectal dynamic contrast enhanced ultrasound

An Aplio 500 ultrasound system (Toshiba Medical, Japan) equipped with a 5–9 MHz transrectal endscan probe with contrast function and VueBox analysis software was applied for TDCE-US examination. The prostate gland and peripheral area were thoroughly scanned to locate the tumor lesion. After location, commercial sulfur hexafluoride (SF6) microbubble (SonoVue, Bracco, Italy) was injected into median cubital vein of the patients. During the examination, the scanning planes with the most abundant blood supply or abnormal nodules were selected as the observation ones, and were dynamically observed for further analysis.

### Magnetic resonance imaging examination

All patients underwent magnetic resonance imaging examination on a 3 T Achieva scanner (Philips Healthcare, Best, Netherlands) applying an endorectal coil (BPX-30, Medrad, Pittsburgh, PA). According to PI-RADS criteria, the main scanning range included the prostate and seminal vesicles. The overall sequences included T2-weighted images (T2WI), T1-weighted images (T1WI), dynamical contrast enhanced (DCE) and diffusion-weighted images (DWI) with high b-value of 1500 s/mm2. Each sequence applied a five-point assessment scale (except for DCE) which graded the level of suspicion for the presence of PCa from 1 to 5 (very low to very high) according to the PI-RADSv2 criteria. T1WI images were obtained with repetition time/echo time (TR/TE) of 400 and 10 ms, a slice thickness 3 mm, and field of view (FOV) of 180 × 180 mm. T2WI images were obtained with TR/TE of 3000 and 90 ms, a slice thickness 3 mm, and field of view (FOV) of 200 × 200 mm. DWI images were obtained with TR/TE of 6000 and 80 ms, a slice thickness 3 mm, and field of view (FOV) of 220 × 220 mm. DCE images were obtained with TR/TE of 4.0 and 1.5 ms, a slice thickness 3 mm, and field of view (FOV) of 220 × 220 mm. All MRI scans were performed and interpreted by two radiologists with more than ten years’ MRI diagnostic experience in our institution. Both of the two radiologists were blinded to the information of patients, and reviewed the MRI images independently, if there was, any discrepancy, they compared the findings and made the diagnosis in consensus.

### Quantitative analysis of TDCE-US

**Figure 1:**
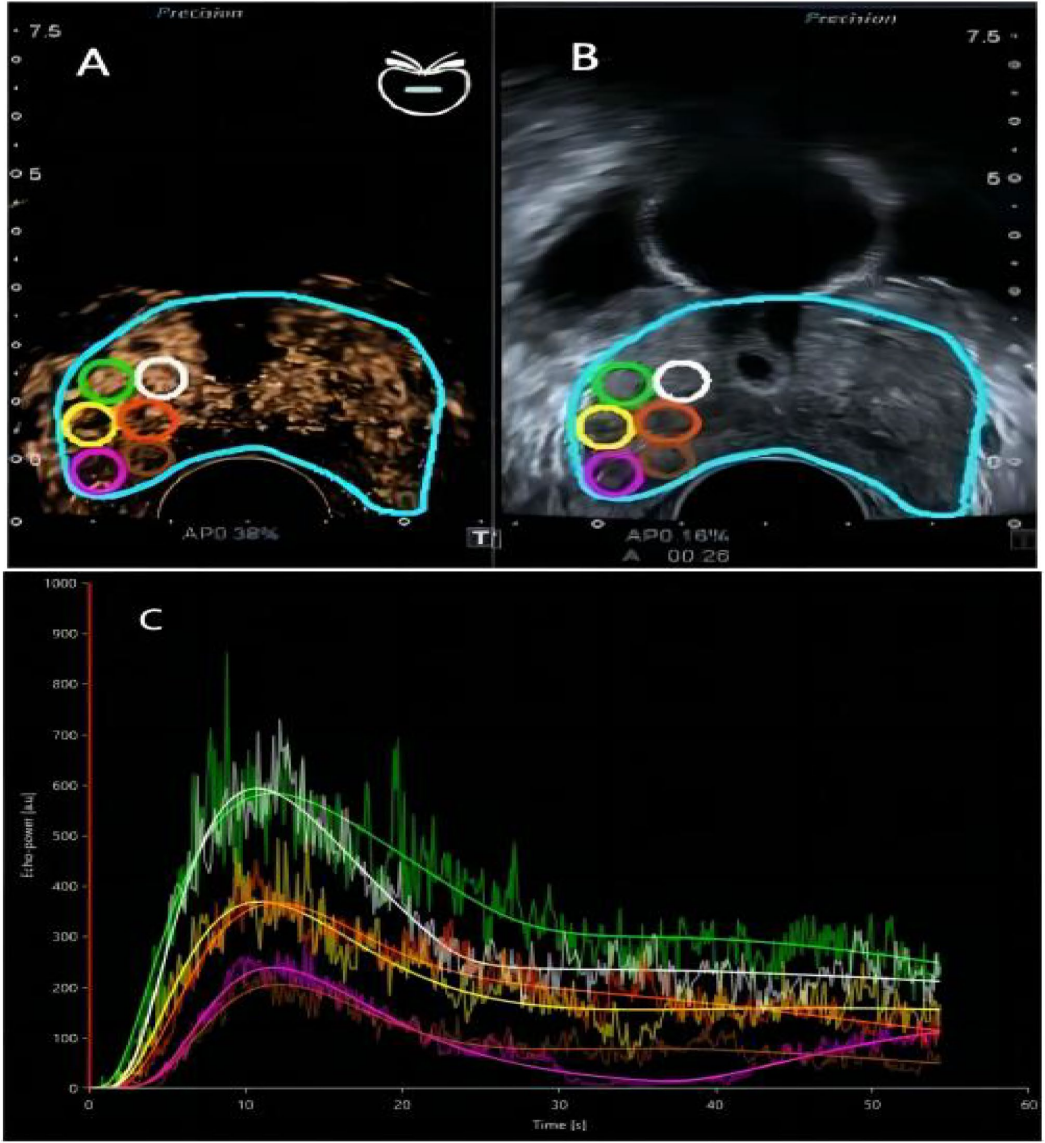
(**A**) Represents the process of outlining ROI in the CEUS imagin mode; (**B**) represents the synchronized picture of A in the gray-scale imaging mode; (**C**) represents the results of Time intensity curve analysis. The process of quantitative analysis: six parts of the contrast-enhanced ultrasound (CEUS) imaging outlined according to systematic biopsy (halfway of the entire process, limited by the ability of analysis software) with quantification by time intensity curve analysis (TIC) after bolus injection of contrast agent. The highest pathological grade located in the lateral border of peripheral zone in the right lobe of prostate, the Gleason score = 4 + 5, the RT = 7.22s, WoAUC = 4330, QOF = 87%, ROI area = 0.21 cm^2^_._

Two radiologists with abundant experience in contrast enhanced ultrasound operation used the VueBox® (Bracco, Italy) software to analyze the cine loops data obtained in TDCE-US examination. The analytic procedures included outlining all the twelve areas of the systematic biopsy in the contrast enhanced ultrasonic imaging with the quality of fit > 75%, and determining the region of interest (ROI), which is defined as the area of core with highest grade in the pathological results following systematic biopsy, then analyzing the ROI area and interpreting into quantitative parameters, the software automatically generated CEUS time-intensity curves, the process is presented in Fig. 1. Consequently, all the following contrast parameters of the ROI were collected from the quantification toolbox: rise time (RT) [s]; time to peak (TTP) [s]; peak enhancement (PE) [a.u]; wash-in rate (WiR) [a.u]; wash-in area under the curve (WiAUC) [a.u];(wash-in perfusion index (WiPI) (WiAUC/RT) [a.u]; mean transit time local (mTTl) [s]; fall time (FT) (TO-TTP) [s]; wash-out AUC (WoAUC) (TTP/TO) [a.u]; [a.u]; (wash-out rate) (WoR) [a.u]; wash-in and wash-out AUC (WiWoAUC) (WiAUC + WoAUC).

### Statistical analysis

The continuous variables were evaluated using Kolmogorov–Smirnov test for normal distribution, The continuous variables with normal distribution were presented as means ± standard deviation and analyzed using Student’s t-test, the skewed distribution ones were presented as medians (ranges) and analyzed using the Mann–Whitney U-test.

Univariate and multivariate analyses were performed to determine independent risk factors strongly associated with csPCa, those variables in the univariate analysis with a *P* value < 0.05 were included in the following multivariate Logistic regression analysis, and the variables in the multivariate Logistic regression analysis with a *P* value < 0.05 were defined as independent risk factors, and the odds ratio (OR) and 95% confidence interval (CI) were determined, and the clinical predictive model 1 was constructed on the basis of independent risk factors excluding TDCE-US parameters, the clinical predictive model 2 was constructed based on all the independent risk factors.

To construct a nomogram with good calibration and discrimination for predicting csPCa, the model ought to be developed in a training set and validated in a validation data set from the same cohorts. All the 151 patients were randomly divided into train cohort (n = 99) and validation cohort (n = 52) with the ratio of 2:1. The nomogram was constructed on the basis of the clinical predictive model 2, the discriminative performance of the nomogram was quantified by the area under the receiver operating characteristic curve (AUROC), and the calibration of the nomogram was assessed using Hosmer goodness-of-fit test and plotted graphically. Comparative analysis between model 1 and model 2 was conducted using Delong’ test^19^ to focus on the AUROC, and as a complementary method to ROC analysis by Pencina et al.^20^, net reclassification improvement (NRI) analysis was conducted to evaluate whether the nomogram of TDCE-US based model 2 could improve the classification of patients compared with model 1. Moreover, the clinical utility of the TDCE-US based model 2 and model 1 is evaluated by decision curve analysis described by Vickers et al.^21^.

Statistical analysis was conducted using R package version 4.1.3 (http://www.R-project.org). The R code of statistical analysis was presented in Supplementary file 1. And all *P* values were two-sided. A *P* value of less than 0.05 was regarded as statistically significant.

### Ethics approval

The trial was conducted in accordance with the Declaration of Helsinki (as revised in 2013). The study was approved by the Chongqing University Cancer Hospital Ethics Committee (No. 2019 (177)) and informed consent was taken from all individual participants. The authors are accountable for all aspects of the work in ensuring that questions related to the accuracy or integrity of any part of the work are appropriately investigated and resolved.

## Results

### Clinical characteristics of the patients

**Figure 2:**
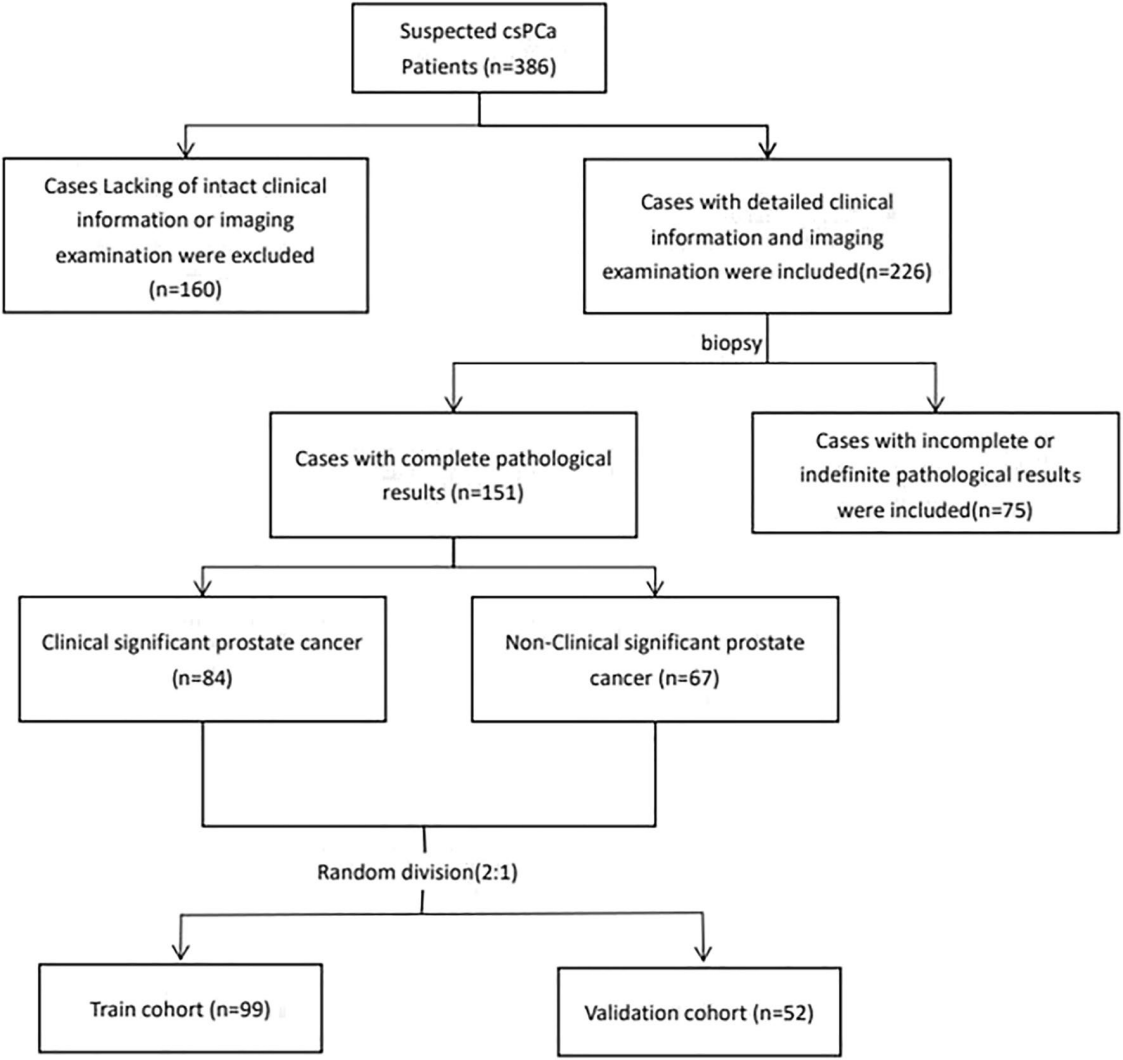
The flowchart outlining patient selection. csPCa, clinically significant prostate cancer.

From 2019 January to 2022 March, 386 patients suspected clinical significant prostate cancer were recorded in the patients record system. Through careful examination, 160 patients with incomplete clinical information or lack of mp-MRI and TDCE-US scanning were excluded, and 75 of the patients left were not definite pathologically. Finally, 151 patients were recruited in this study (84 patients were csPCa and 67 patients were Non-csPCa). The flowchart of selection is shown in Fig. 2. Using the random split method, 99 patients were divided into train cohort, 52 patients were divided into validation cohort, and the csPCa prevalence between the train cohort and validation cohort was not significantly different (53.5% vs 59.6%, *P* = 0.625). There was no significant difference appeared in age, PSAD, TPSA, FPSA, f/t, PI-RADS, and all the TDCE-US parameters (Table 1) between train cohort and validation cohort. And the fundamental characteristics of the two cohorts are presented in Table 1.

**Table 1:** Characteristics of the patients in training/validation cohort, csPCa/Non-csPCa group.

Characteristics	Training cohort	Validation cohort	*P*	csPCa	Non-csPCa	P*
Age (year)	71.45 ± 6.87	72.50 ± 5.98	0.62	72.60 ± 5.82	68.31 ± 7.08	0.006
BMI (kg/m^2^)	23.86 ± 2.63	23.77 ± 2.31	0.72	23.74 ± 2.51	23.91 ± 2.36	0.623
PV (cm^3^)	41.2 ± 4.5	42.3 ± 4.6	0.82	36.9 ± 4.09	46.5 ± 5.23	< 0.001
TPSA (ng/mL)	20.00 (4.73, 73.86)	15.27 (4.83, 52.03)	0.92	20.57 (3.51, 109.90)	13.60 (1.59, 29.23)	0.002
FPSA (ng/mL)	1.78 (1.11, 3.80)	1.24 (1.09, 3.34)	0.31	2.82 (1.65, 17.22)	2.29 (1.44, 11.53)	0.015
f/t	0.16 (0.12, 0.18)	0.16 (0.11, 0.21)	0.79	0.13 (0.08, 0.18)	0.17 (0.13, 0.20)	0.373
PSAD (ng/mL/cm^3^)	0.85 (0.38, 1.07)	0.71 (0.30, 098)	0.68	1.40 (0.57, 2.30)	0.44 (0.18, 0.99)	< 0.001
WiAUC	2324 (1348, 4773)	2097 (1251, 4153)	0.38	2329 (1312, 4309)	2151 (1232, 5059)	0.192
mTTls	57.40 (38.31, 87.72)	44.09 (35.42, 87.98)	0.29	45.31 (34.12, 74.31)	65.29 (43.99, 130.29)	0.025
WiR	61.59 (32.10, 145.52)	41.14 (20.82, 79.42)	0.12	64.80 (32.84, 144.19)	46.20 (19.10, 88.00)	0.413
WiPI	247.9 (148.7, 460.9)	199.7 (122.3, 347.3)	0.24	230.5 (154.9, 230.5)	244.3 (99.7, 351.5)	0.357
WiWoAUC	7159 (4595, 16,593)	6012 (3833, 12,709)	0.42	6972 (4532, 15,509)	6471 (4219, 18,392)	0.117
TTPs	14.42 (11.54, 19.05)	14.31 (10.93, 18.78)	0.71	13.10 (10.36, 17.64)	15.09 (11.86, 23.01)	0.010
RTs	9.42 (7.13, 12.36)	9.19 (6.43, 13.38)	0.89	7.57 (6.15, 9.94)	12.21 (9.42, 17.32)	< 0.001
WoR	20.80 (8.91, 46.58)	15.26 (7.08, 28.40)	0.18	19.37 (8.92, 55.47)	16.15 (7.11, 32.68)	0.420
PE	381.8 (236.7, 700.1)	321.9 (234.7, 591.4)	0.41	343.1 (237.5, 696.7)	370.8 (210.5, 651.1)	0.434
FTs	23.20 (15.42, 32.45)	19.12 (14.81, 29.83)	0.29	20.99 (14.69, 30.61)	23.42 (15.78, 32.81)	0.876
WoAUC	8647 (3847, 12,762)	5301 (2788, 11,297)	0.11	5161 (3007, 10,697)	10,585 (4099, 15,277)	0.001
PI-RADS score			0.52			< 0.001
1	29 (29.3%)	14 (26.9%)		11 (13.1%)	32 (47.8%)	
2	12 (12.1%)	9 (17.3%)		7 (8.3%)	14 (20.9%)	
3	11 (11.1%)	9 (17.3%)		13 (15.5%)	7 (10.4%)	
4	28 (28.3%)	11 (21.2%)		29 (34.5%)	10 (14.9%)	
5	19 (19.2%)	9 (17.3%)		24 (28.6%)	4 (6.0%)	

*P* is the probability of the difference between training cohort and validation cohort. P*is the probability of the difference between csPCa and Non-csPCa cohort.

TPSA, total prostate-specific antigen; FPSA, free-prostate specific antigen; f/t, free-to-total prostate-specific antigen ratio; PSAD, prostate-specific antigen density; WiAUC, wash in area under the curve; mTTls, mean transit time local(s); WiR, wash-in rate; WiPI, wash-in perfusion index; WiWoAUC, wash-in and wash-out AUC; TTPs, time to peak(s); RTs, rush time(s); WoR, wash-out rate; PE, peak enhancement; FTs, fall time(s); WoAUC, wash-out area under the curve; PI-RADS, prostate imaging reporting and data system.

### Univariate and multivariate analysis

Univariate analysis in the train cohort indicated that the indicators including Age, TPSA, FPSA, PSAD, mTTl, TTP, RT, WoAUC, PI-RADS score were significantly different between the csPCa and non-csPCa groups, (*P* < 0.05, Table 1). Then, these indicators were included in the multivariate regression analysis, and the result showed that Age, PSAD, RT, WoAUC and PI-RADS score were independent predictors in the detection of csPCa (*P* < 0.05, Table 2). As a consequence, the predictive model 1 finally integrated three clinical factors: Age, PSAD and PI-RADS score. And then the TDCE-US parameters RT and WoAUC were added into model 1 to develop a new predictive model 2.

**Table 2:** Multivariate regression analysis in the training cohort.

Variables	β	Odds Ratio	95% CI	*P* value
Age	0.100	1.106	0.983, 1.265	0.029
PV	0.358	1.431	1.102, 2.231	0.074
TPSA	0.333	1.396	1.086, 2.016	0.127
FPSA	0.279	1.322	1.012, 1.953	0.183
PSAD	0.505	1.658	1.169, 2.522	0.007
mTTls	− 0.110	0.896	0.769, 0.982	0.230
TTPs	− 0.080	0.923	0.782, 0.993	0.132
RT	− 0.143	0.866	0.750, 0.973	0.031
WoAUC	− 0.164	0.849	0.733, 0.954	0.014
PI-RADS score				
2	2.387	10.88	1.496, 104.9	0.024
3	2.542	12.70	1.541, 148.1	0.025
4	3.882	48.55	7.097, 550.1	< 0.001
5	4.023	55.86	7.458, 810.6	< 0.001

PSAD, prostate-specific antigen density; RTs, rush time(s); WoAUC, wash-out area under the curve; PI-RADS, prostate imaging reporting and data system.

### Nomogram

**Figure 3:**
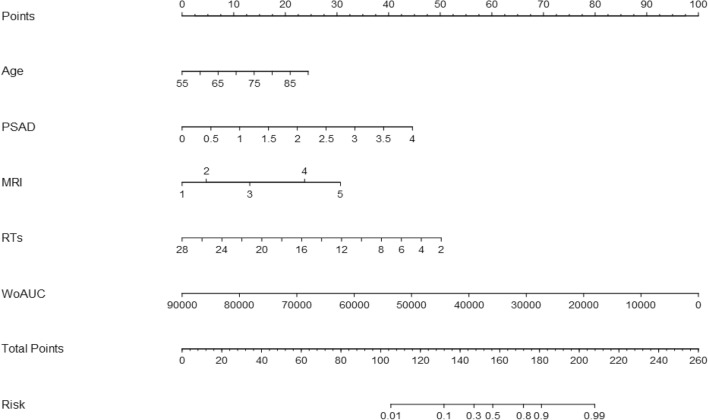
Constructed nomogram based on transrectal dynamic contrasted enhanced ultrasound, PI-RADS score, PSAD and age. PSAD, prostate-specific antigen density; MRI, magnetic resonance imaging; RTs, rush time(s); WoAUC, wash out area under the curve.

On the basis of model 2, the individualized predictive nomogram was constructed (Fig. 3). The top row showed the point assignment for each variable. Rows 2 to 6 represented the indicators included in the nomogram. For an individual patient, each indicator was assigned a definite point according to the characteristics. The points assigned to each of the six variables were added, and the total points were presented in row 7. The total points in row 7 corresponded to the risk of csPCa in row 8.

### Evaluation and comparison of the predictive models

**Figure 4:**
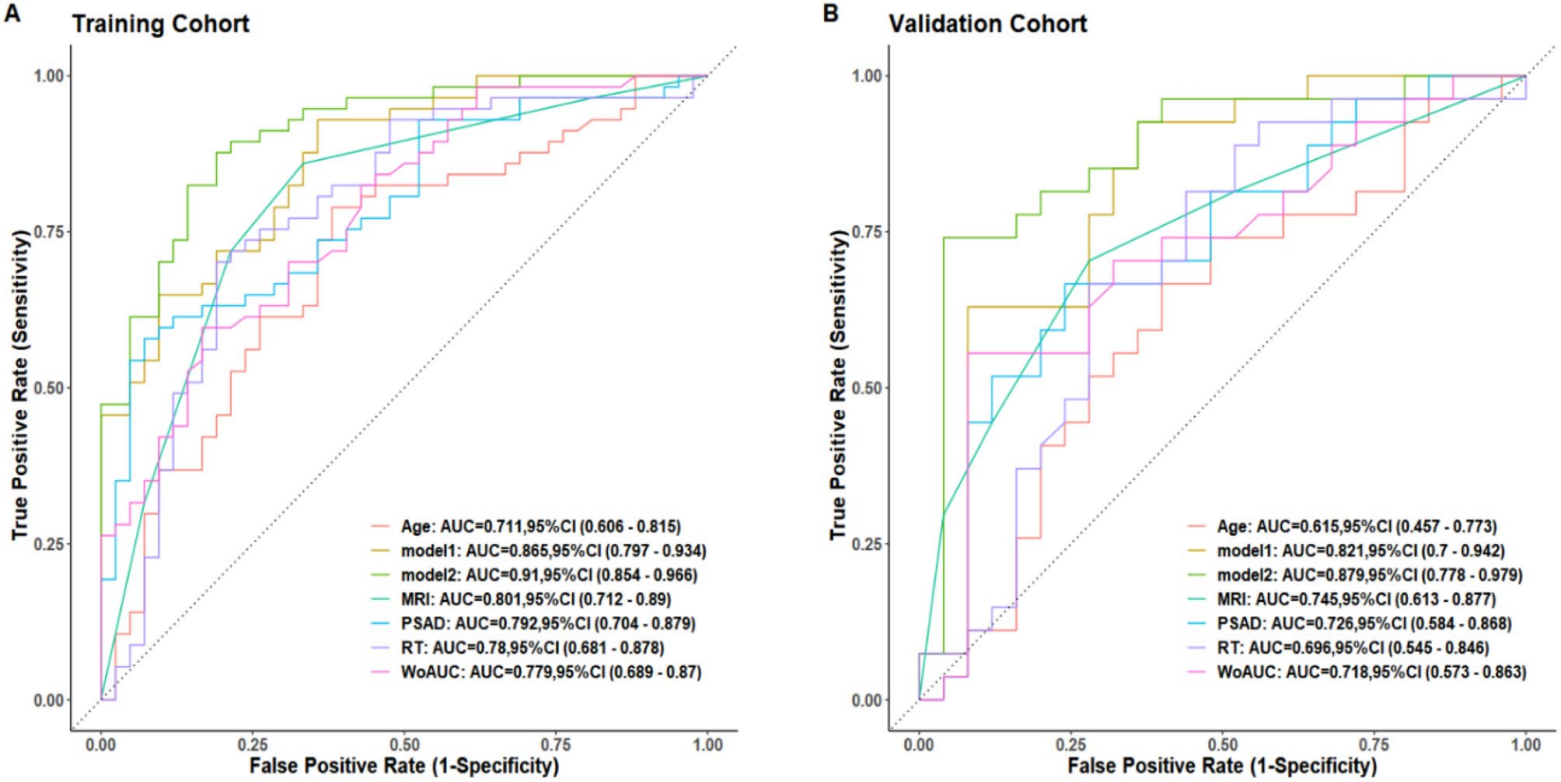
AUROC curve analysis in the training cohort (**A**), and validation cohort (**B**). PSAD, prostate-specific antigen density; MRI, magnetic resonance imaging; RT, rush time(s); WoAUC, wash out area under the curve.

The discriminative performances of the models were evaluated using AUROC curves. In the train cohort, the AUROC of model 1 was 0.865 (95% CI 0.797, 0.934),and the AUROC of the model 2 was 0.910 (95% CI 0.854, 0.966), Delong’ test showed that the difference of AUROC was significant (z = 1.98, *P* = 0.04). In the validation cohort, the AUROC of model 1 was 0.821 (95% CI 0.700, 0.942), and the AUROC of model 2 was 0.879 (95% CI 0.778, 0.979), Delong’ test showed that the difference of AUROC was significant (z = 2.02, *P* = 0.02), all the AUROC were presented in Fig. 4. In the train cohort, using a cut-off value of 0.559, the sensitivity of model 2 was 81.0%, and specificity was 87.7%, using a cut-off value of 0.289, the sensitivity of model 1 was 64.3%, and specificity was 93.0%. In the validation cohort, using a cut-off value of 0.447, the sensitivity of model 2 was 83.3%, and specificity was 87.7%, using a cut-off value of 0.599, the sensitivity of model 1 was 83.3%, and specificity was 71.9%. The results above showed that the discriminative value of csPCa of model 2 was better than model 1.

Moreover, NRI analysis indicated that the nomogram based on model 2 had improved patient classification in both the train cohort (NRI = 0.114) and validation cohort (NRI = 0.158) compared to model 1.

**Figure 5:**
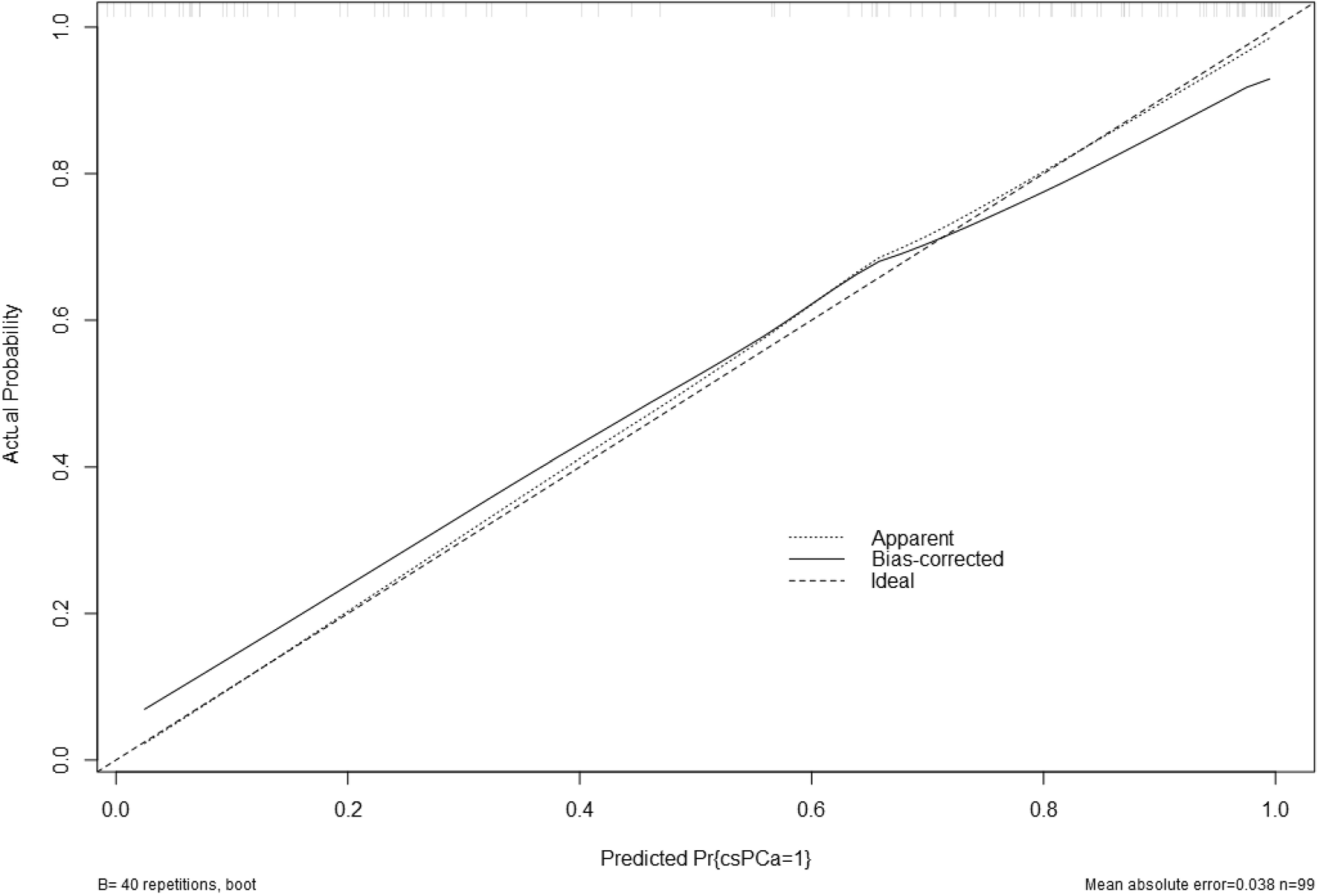
The calibration curve of the nomogram in the training cohort.

The calibration curve of the nomogram based on model 2 showed a good agreement between the predicted probability and the actual risk of csPCa (Fig. 5). The Hosmer–Lemeshow goodness-of-fittest also indicated that the nomogram is well fitted (*P* = 1.126).

**Figure 6:**
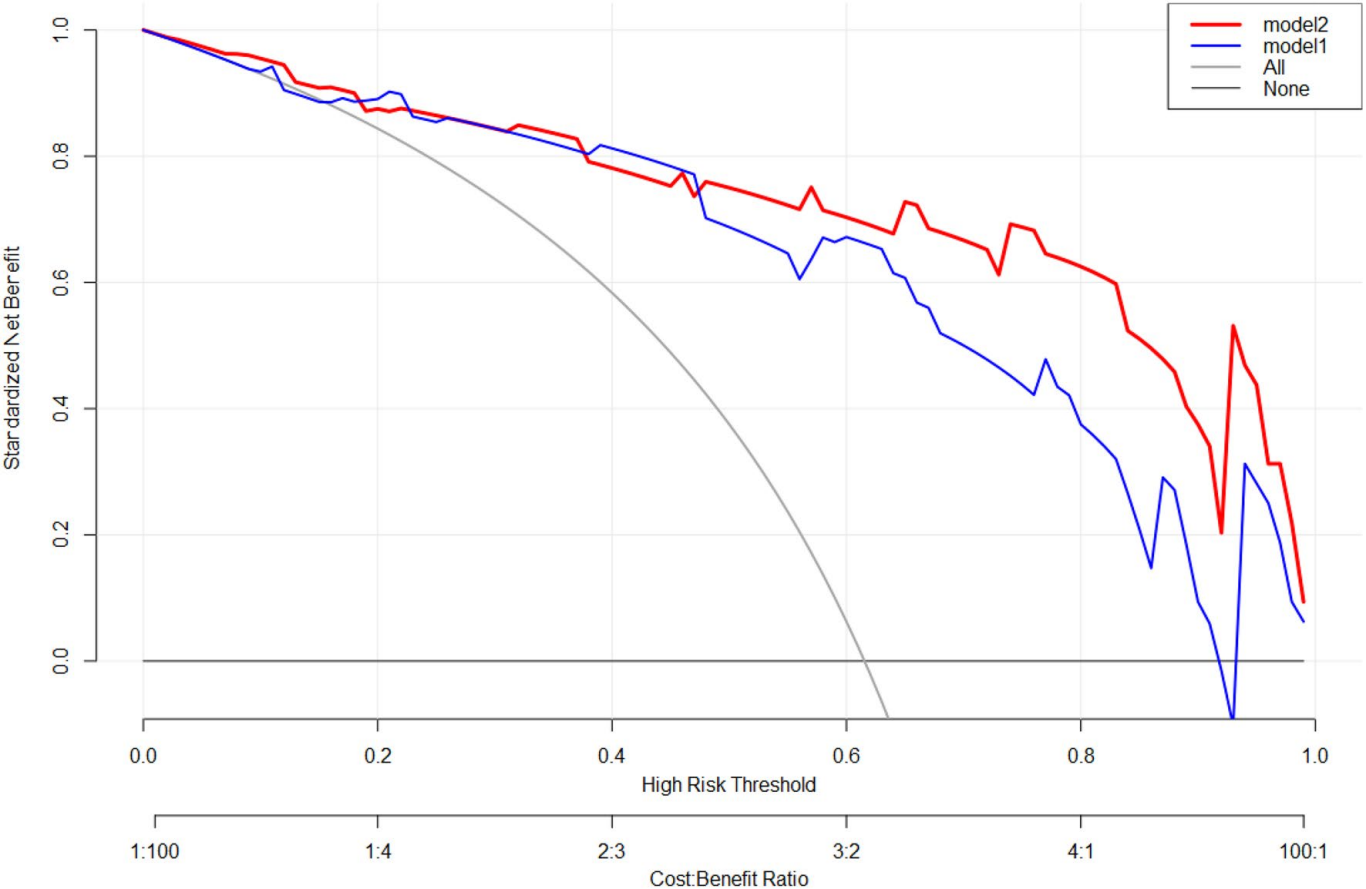
Decision curve analysis in the validation cohort.

The decision curve analysis is presented in Fig. 6.When the threshold probability is greater than 0.48, the net benefit of applying the nomogram based on model 2 was much higher than that of the model 1, and when the threshold probability is less than 0.48, the difference of net benefit between the two models is not obvious. The result suggested that this nomogram based on model 2 is better in guiding the decision-making in clinical practice.

## Discussion

Through interpreting the mp-MRI scanning results of prostate into definite numerical levels, PI-RADSv2 score has become a primary diagnostic modality to differentiate benign prostatic lesions from malignancy. Some studies^22,23^ have shown excellent diagnostic performance of PI-RADSv2 score. Nevertheless, there are some limitations of PI-RADSv2 score, the primary controversy is the intra-observer disagreement,

Muller et al.^24^ found a sensitivity of 85–88% for diagnosing clinically significant prostate cancer using PIRADSv2 score, but a kappa score of 0.46 existed between observers, another influential factor could lower diagnostic sensitivity is that some benign lesions such as prostatitis, high-grade prostatic intraepithelial neoplasia, and hyperplasia would behave as PCa pathologically, which would increase the misdiagnostic rate of prostate cancer^25^. Using PI-RADSv2 score alone would not help improve diagnostic accuracy of prostate cancer and reduce overtreatment and biopsy. Different approaches incorporating PI-RADSv2 score and other diagnostic imaging or clinical laboratory indicators emerged in recent years for risk stratification of prostate cancer. Wang^26^ et al. combined PI-RADS score with PSAD to detect clinically significant prostate cancer, the specificity and positive predictive values of which were 93.5% and 82.4%, respectively. Ding^27^ et al. combined PI-RADSv2 score and ultrasonic elastography-derived data to stratify prostate cancer, and found that the nomogram they constructed combing elastography-derived and mp-MRI data was more clinically useful than the model based on PI-RADS score and clinical parameters alone.

Another imaging method may help improve diagnostic accuracy and clinical utility is CEUS, which is a backscattered ultrasonic signal-based examination that can be applied to detect and characterize prostate cancer by displaying the microbubble in the blood perfusion of tumor and surrounding tissue. However, combination of CEUS quantitative parameters, PI-RADS and clinical parameters to predict csPCa has rarely been reported.

In this retrospective study, we established and validated a novel nomogram combining quantitative CEUS parameters (qCEUS), PI-RADsv2 score and clinical parameters for the prediction of suspected csPCa before biopsy or medication is performed. The nomogram achieved satisfactory discrimination, classification and calibration performance for csPCa prediction.

The nomogram was convenient to use in clinical practice with six predictive items: age of the patient, PI-RADS score, PSAD, and two TDCE-US quantitative parameters including RT, WoAUC. This nomogram incorporated the advantages from several medical aspects including ultrasound, MRI, clinical indicators, laboratory results, and performed a higher discriminative ability than the model combining only PI-RADS score and clinical parameters. This nomogram allowed for accurate individualized pre-biopsy prediction of csPCa and reduced unnecessary biopsy and treatment.

As a visualized and convenient form of statistical predictive model, nomogram could integrate various predictive indicators from different aspects in one table, and provide an accurate probability of clinical events via presenting a user-friendly interface graphically. Nomogram has been worldwide applied for the risk assessment and prognosis evaluation of disease because of its convenience and accuracy. In this study, we developed a novel nomogram combining transrectal dynamic contrasted enhanced ultrasound, PI-RADS score, PSAD, and patients’ age with AUROCs of 0.910 (95% CI 0.854–0.966) and 0.879 (95% CI 0.778–0.979), in the train cohort and in the validation cohort, respectively. And this is higher significantly than commonly used model combining patients’ age, PSAD, PI-RADSv2 score^28^ showing that the AUROC for predicting csPCa were 0.86 (95% CI 0.79–0.93) and 0.83 (95% CI 0.76–0.89) in the train cohort and validation cohort. But the AUROC was lower than that reported in another study incorporating PI-RADSv2 score and PSAD^29^, which reported a high AUROC of 0.936 in the training cohort and 0.963 in the validation cohort in the transition zone. Though the discriminative performance of the model in our study was not that high, we did incorporated quantitative ultrasound parameters, a totally different imaging modality into PI-RADSv2, and improved the performance of PI-RADSv2 score in this study’s patients. This study proved that quantitative contrast enhanced ultrasound could help provide more useful information when radiologists or clinicians evaluate prostatic lesions using PI-RADSv2 criteria.

After discriminative ability and clinical utility-based statistical analysis, TDCE-US quantitative parameters combined with PI-RADS score improved the diagnostic accuracy and clinical utility more significantly compared to using PI-RADS score alone. Additionally, this nomogram made an economic progress in improving the ability of PI-RADS score in detecting csPCa. TDCE-US is more economical than dynamic contrast-enhanced and diffusion MRI. According to a recently published study^10^, quantitative apparent diffusion coefficient (ADC) value is helpful in improving risk stratification of PI-RADS score.

The predictive variables in the nomogram are all independent factors obtained by multivariate regression analysis, only two clinical variables incorporated in this nomogram, TPSA, FPSA, f/t were not included for *P* value greater than 0.05, though they were important laboratory indicators of csPCa. Before performing the analysis, we excluded digital rectal examination (DRE), another important clinical indicator had been proposed by many previous studies^30,31^ in the detection of csPCa. Because DRE is usually a kind of subjective examination, the detection rate of lesion is relative to the experience of the doctors, if the nomogram incorporates DRE, the reliability may be affected, meanwhile, the aim of constructing this nomogram is to facilitate the diagnostic process, DRE may increase the burden of assessment in some degree.

This study has several limitations. Firstly, this was a retrospective study performed at a cancer hospital, many patients were admitted to hospital with clinical symptoms. So a selection bias is inevitable, about 55.6% of the patients in this study are csPCa, which is higher than the normal occurrence rate of csPCa. Secondly, the number of patients in this study was small, large-sample randomized studies are required for convincing results. Thirdly, this was a single-center study, there was no external validation in other hospitals to inspect the diagnostic stability of this nomogram.

## Conclusion

In conclusion, this study constructed and validated a novel nomogram integrating transrectal dynamic contrasted enhanced ultrasound, PI-RADS score, PSAD and patients’ age. This is an accurate and convenient predictive model, which is helpful to improve the diagnostic ability and clinical utility of PI-RADS score in clinically significant prostate cancer. Quantitative CEUS may be taken into consideration when PI-RADS criteria update for the next version.

### Supplementary Information

Supplementary Information.

## Data Availability

The datasets generated and analysed during the current study are not publicly available due to further relevant studies applying the data are processing and our institution forbids uploading any patients’ private data in public. But part of the datasets are available from the corresponding author on reasonable request in future.

## Abbreviations

TDCE-US: Transrectal dynamic contrast-enhanced ultrasound
csPCa: Clinically significant prostate cancer
PI-RADS: Prostate imaging reporting and data system
Mp-MRI: Multi-parameter magnetic resonance imaging
PSAD: Prostate-specific antigen density
TPSA: Total prostate-specific antigen
FPSA: Free prostate-specific antigen
f/t: Free-to-total prostate-specific antigen ratio
ADC: Apparent diffusion coefficient
RT: Rush time
WoAUC: Wash-out area under the curve
WiAUC: Wash in area under the curve
mTTls: Mean transit time local(s)
WiR: Wash-in rate
WiPI: Wash-in perfusion index
WiWoAUC: Wash-in and wash-out area under the curve
TTPs: Time to peak(s)
WoR: Wash-out rate
PE: Peak enhancement
FTs: Fall time(s)
ROI: Region of interest
