# Supplementary material for: A novel model incorporating quantitative contrast-enhanced ultrasound into PI-RADSv2-based nomogram detecting clinically significant prostate cancer
Source: Sci Rep. 2024 May 15;14:11083. doi: 10.1038/s41598-024-61866-x (PMC11093975; doi:10.1038/s41598-024-61866-x)
Supplement: Supplementary file 1 — Supplementary Information. [file 41598_2024_61866_MOESM1_ESM.pdf]

```
rm(list = ls())
install.packages('VIM')
install.packages('naniar')
install.packages('rlang')
install.packages('mice')
#install.packages("devtools")
devtools::install_github(repo = "amices/mice")
library(rlang)
library(VIM)
library(naniar)
library(ggplot2)
library(mice)
data<- read.csv('C:/Users//Desktop/data.csv')
summary(data)
data <- data
nrow(data)
data$MRI <- as.factor(data$MRI)
summary(data)
varlist<-
c("FPSA","FT","PSAD","WiAUC","mTTls","WiR","WiPI","WiWoAUC","TTPs","WoR","PEa.u","FTs",
                         "Age","TPSA","MRI","RTs","WoAUC","csPCa")

data.impu = data[varlist]
impu_norm.boot <- mice(data.impu, maxit = 0)
summary(impu_norm.boot)
pred <- quickpred(data.impu)
pred
summary(data)
meth<- impu_norm.boot$meth
meth
K<-20
data_imputated <- vector(K,mode="list")


imputation_50 <- mice(data.impu, maxit = 25, m = K, seed = 1234, pred = pred, meth = meth,
print = TRUE)

for (i in 1:K) {
   data_imputated[[i]] <- mice::complete(imputation_50, i)
}
summary(data_imputated[[i]])
nrow(data_imputated[[i]])
data_multiple<-data_imputated[[i]]
summary(data_multiple)
```

```
prop_train <- 0.66

train <- sample(nrow(data_multiple), nrow(data_multiple) * prop_train)

data.train <- data_multiple[train,]
data.test <- data_multiple[-train,]
summary(data.train)
summary(data.test)
nrow(data.train)
nrow(data.test)

install.packages("autoReg")
library("autoReg")
overall.log<-glm(csPCa~Age+BMI+PV+TPSA+FPSA+f/t+PSAD+WiAUC+mTTls+WiR+WiPI+WiWoAU
C+TTPs+RTs+WoR+PE+FTs+WoAUC+PI-RADS ,data=train,family=binomial)
summary(overall.log)
model1<-autoReg(overall.log,uni=TRUE,multi=FALSE,threshold=0.05)
model1
model2<-autoReg(overall.log,uni=FALSE,milti=TRUE,threshold=0.05)
model2
model3<-autoReg(overall.log,uni=TRUE,milti=TRUE,threshold=0.05)
model3

install.packages("pROC")
install.packages("plotROC")
library(pROC)
library(plotROC)

Outcome <- "csPCa"
FinalVariables1 <- c("Age","PSAD","PI-RADS","RT","WoAUC")
Formula <- formula(paste(paste(Outcome,"~", collapse=" "),
                         paste(FinalVariables1, collapse=" + ")))
model.finaltest1 <- glm(Formula, data=data.train,family=binomial)
summary(model.finaltest1)

abc <- predict(model.finaltest1, data.train, type="response")

a <- roc(data.train$csPCa, abc)

c_1<- a$auc

Outcome <- "csPCa"
```

```
FinalVariables3 <- c("Age","PSAD","PI-RADS")
Formula <- formula(paste(paste(Outcome,"~", collapse=" "),
                         paste(FinalVariables3, collapse=" + ")))
model.finaltest3 <- glm(Formula, data=data.train,family=binomial)
summary(model.finaltest1)

cde <- predict(model.finaltest3, data.train, type="response")

c <- roc(data.train$csPCa, cde)

c_3<- c$auc



Outcome <- "csPCa"
FinalVariables2 <- c("Age","PSAD","PI-RADS")
Formula <- formula(paste(paste(Outcome,"~", collapse=" "),
                         paste(FinalVariables2, collapse=" + ")))
model.finaltest2 <- glm(Formula, data=data.test,family=binomial)
summary(model.finaltest2)

bcd <- predict(model.finaltest2, data.test, type="response")

b <- roc(data.test$csPCa, bcd)

c_2 <- b$auc

Outcome <- "csPCa"
FinalVariables4 <- c("Age","PSAD","PI-RADS","RT","WoAUC")
Formula <- formula(paste(paste(Outcome,"~", collapse=" "),
                         paste(FinalVariables4, collapse=" + ")))
model.finaltest4 <- glm(Formula, data=data.test,family=binomial)
summary(model.finaltest4)

def <- predict(model.finaltest4, data.test, type="response")

d <- roc(data.test$csPCa, def)

c_4<- d$auc


roc.test(b,d,method='delong')
roc.test(a,c,method='delong')
```

```
dd <- datadist(data.train)
options(datadist="dd")
fit2<- lrm(csPCa~ Age+PSAD+PI-RADS+RT+WoAUC,
           data=data.train)
nomogram <- nomogram(fit2,fun=function(x)1/(1+exp(-x)),
                     fun.at = c(0.01,0.1,0.3,0.5,0.8,0.9,0.99),
                     funlabel = "Risk",
                     lp=F,
                     conf.int = F,
                     abbrev = F
)

plot(nomogram)

install.packages("rmda")
library(rmda)
data.train$csPCa<-as.numeric(data.train$csPCa)
data.train$csPCa <- as.factor(data.train$csPCa)
fit1 <- decision_curve(csPCa~ Age+PSAD+MRI+RTs+WoAUC,
                       data =data.test,
                       study.design = "cohort",
                       bootstraps = 50
)
fit2 <- decision_curve(csPCa~ Age+PSAD+MRI,
                       data =data.test,
                       study.design = "cohort",
                       bootstraps = 50
)
plot_decision_curve(list(fit1, fit2),
                    curve.names = c("model2", "model1"),
                    xlim = c(0, 1),
                    legend.position = "topright",
                    col = c("red","blue"),
                    confidence.intervals = "none",
                    lty = c(1,1),
                    lwd = c(3,2,2,1)
)
plot_clinical_impact(fit1,
                     population.size= 1000,
                     cost.benefit.axis = T,
                     n.cost.benefits= 8,
                     col=c('red','blue'),
                     confidence.intervals= T,
                     ylim=c(0,1000),
```

```
legend.position="topright",
)
```
